# The Influence of Ectoine on the Skin Parameters Damaged by a CO_2_ Laser

**DOI:** 10.3390/molecules30112470

**Published:** 2025-06-05

**Authors:** Izabela Załęska, Urszula Goik, Tomasz Goik, Kinga Wilkus

**Affiliations:** 1 Academy of Applied Sciences in Nowy Targ, 34-400 Nowy Targ, Poland; 2 Department of Engineering and Machinery for Food Industry, Faculty of Food Technology, University of Agriculture in Krakow, 30-149 Kraków, Poland; 3 Department of Applied Mechanics and Biomechanics, Faculty of Mechanical Engineering, Cracow University of Technology, 31-864 Kraków, Poland

**Keywords:** ectoine, skin, laser, skin treatment

## Abstract

Ectoine is a substance produced by extremophiles and is naturally used by them as protection against adverse environmental conditions in which they live. Scientific contributions discuss its excellent effect through cosmotropic properties, prevention of secondary messenger release in cells, and transcription factors. The influence on the lipid layer of the cell membrane and its preventive effect as a UV filter were also demonstrated. What is more, its anti-oxidative effect was established. Ectoine works as an immunostimulant and also has anti-inflammatory and anti-cancer properties. These attributes are dominating factors in the use of ectoine’s properties in skin fractionation treatment with a C$O_{2}$ laser. In the following work, the influence of ectoine on skin parameters was described, focusing on redness, moisturization, and TEWL after the use of a C$O_{2}$ laser (Załęska 2019). The rheological properties of preparations with ectoine addition were also tested. The yield point was determined, the viscosity changes of cosmetic preparations were measured with increasing shear rates, and oscillation tests were performed. With increasing percentages of ectoine and frequency of application, the occurrence of redness after C$O_{2}$ therapy decreased. The highest moisture level values from 54.4 × 0.02 mg/c$m^{2}$ to 72.5 × 0.02 mg/c$m^{2}$ were obtained for preparation A applied twice a day; for the same preparation, a reduction in TEWL from 6.2 to 5.3 g/($m^{2}$·h) was obtained. The results of the tests of cosmetic emulsions allowed us to conclude that the preparations in the analyzed shear rate range at all tested temperatures are non-Newtonian liquids that are shear-thinning and have a flow limit. The obtained results of the conducted research prove the positive effect of dermocosmetics with ectoine content in the process of skin healing.

## 1. Introduction

**Figure 1:**
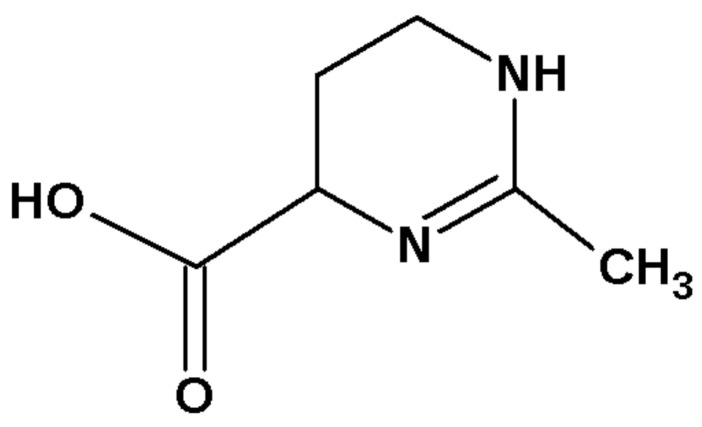
Ectoine structure.

The environment and lifestyle have a direct impact on the shape and condition of the human skin [1]. Diet, environmental pollution, lack of exercise, a sedentary lifestyle, especially in dry and air-conditioned rooms, living in a hurry, stress, and sleep deprivation, as well as frequent exposure of the skin to UV radiation, trigger the generation of Reactive Oxygen Species (ROS), which negatively affect the state of our cells [2]. The human body tries to counteract the negative effects of ROS; healthy skin cells activate defense and repair mechanisms. When cells become insufficient, it is necessary to use substances that prevent and eliminate the negative effects of ROS. Promising raw materials that support skin regeneration are compounds synthesized by extremophilic microorganisms, used by them to protect against the adverse conditions of the environment in which they live [3]. Ectoine is a compound with such properties and it has recently been used in cosmetic formulations. Ectoine is an amino acid, 1,4,5,6-tetrahydro-2-methyl-4-pyrimidinecarboxylic acid (Figure 1), naturally produced by many types of microorganisms to protect cells and their organelles from the adverse effects of the external environment [4,5,6]. Microorganisms living, for example, in geysers, salt lakes, and deserts produce ectoine—which protects them from harmful factors such as high temperature, very low humidity or UV radiation. It appears commonly in aerobic, chemoheterotrophic and halophilic organisms and enables them to survive in extreme conditions [3].

Ectoine was isolated for the first time from extremophilic, halophilic and phototrophic bacteria *Ectothiorhodospira halochloris* and described by Galiński in 1985 [7]. Ectoine belongs to osmoprotectants, which are produced when the osmotic pressure inside the cell is too high, preventing the loss of water from the cell, while not affecting the metabolism of the microorganism even at high concentrations in the cytoplasm. Ectoine not only maintains osmotic activity but also has the ability to protect biomolecules in cells, such as proteins, enzymes and membranes, against dehydration, drying, heating and freezing [8]. The growing demand for ectoine has resulted in increased production of ectoine from microbiological sources. Many microbiological sources of ectoine and its derivatives, as well as microbiological production and fermentation methods, have been characterized. Traditional methods and new technologies for improved production and recovery of ectoine from microbial fermentation are known and used [9]. Ectoine synthesis is initiated by microorganisms in extreme environmental conditions and is inhibited when the stress factor disappears. Ectoine is a kind of defense system for these microorganisms. Ectoine is synthesized in a similar way to other cyclic amino acids. On an industrial scale, ectoine is produced in a complex high-salt process [10]. In [11], a new strain of *Corynebacterium glutamicum* for use in the low-salt fermentation process using well-known raw materials for the production of ectoine with industrial efficiency was proposed. In the next report [12], an ectoine-producing *Escherichia coli* variant, ET08, was constructed by introducing the ectABC gene cluster and eliminating the metabolic pathways involving lysine and pyruvate. Increasing the ectoine production of *E. coli* has great industrial prospects. Ectoine has cosmotropic properties, i.e., it has the ability to bind and incorporate water molecules into complexes. Thanks to this, it helps in the reconstruction of the skin cell membrane [13], moisturizes the skin [14,15], protects the lipid layers of cell membranes, and acts preventively as a filter protecting the skin against UV rays [3,16]. Ectoine prevents various skin diseases such as photocarcinogenesis, photodermatoses, and photoaging by protecting skin cells with its singlet oxygen-quenching properties and delaying the skin aging process, which is often associated with skin exposure to UVA radiation causing the production of singlet oxygen [17]. Ectoine also protects DNA against ionizing radiation [18] and has an antioxidant effect, delaying premature skin aging [16]. Moreover, ectoine has been shown to be useful in the treatment of atopic dermatitis [19,20] and preventing the negative effects of radiotherapy and chemotherapy on the human body [21], and it can also be used as a skin-whitening substance [22]. Ectoine has an immunostimulating effect, and also has anti-inflammatory and anti-cancer properties [23]. Ectoine has found widespread application in the cosmetics industry due to its protective and stabilizing effects on human skin cell membranes against harmful external factors such as ultraviolet (UV) radiation, wind, humidity, and drastic temperatures [24]. In [25] the adverse effects of stress on the skin (damage to keratinocyte proteins, loss of basement membrane proteins, and collagen degradation) were studied. These studies confirm the effectiveness of ectoine in skin regeneration and anti-aging.

Ectoine was also found to improve the dispersion and hydration of keratin bundles in corneocytes to a greater extent than hydration with water alone [26]. The results obtained in [27] show that ectoine can also be a good ingredient for improving the safety of cleansing cosmetics. The C$O_{2}$ laser causes micro-damage to the skin, initiating healing processes such as inflammation, re-epithelialization, and collagen remodeling. This treatment temporarily damages the skin barrier, increases transepidermal water loss (TEWL), and induces local inflammation and oxidative stress, which may lead to discomfort, prolonged redness, and the risk of post-inflammatory hyperpigmentation (PIH). That is why we are looking for various solutions and methods to support skin regeneration processes after laser treatments. Previous methods of supporting skin regeneration include, among others, testing the effects of skincare products with a copper tripeptide complex, platelet-rich plasma, betulin-based emulsions, preparations with *Centella asiatica* extracts, panthenol, and recombinant human epidermal growth factor (rhEGF). The study by [28] assessed redness, overall improvement of wrinkles, and overall improvement of skin appearance after 12 weeks of treatment with a copper tripeptide complex (glycyl-L-histidyl-L-lysine-C$u^{2+}$). The use of skincare products with a copper tripeptide complex after skin resurfacing treatment with a C$O_{2}$ laser did not result in a significant reduction or elimination of post-treatment erythema. The use of platelet-rich plasma on laser-ablated skin to deliver concentrated growth factors to accelerate healing and rejuvenation, as well as to shorten patient recovery time, has also been studied with good results [29]. Laser treatment of skin lesions using an emulsion preparation with betulin addition as an active ingredient has also been studied. In comparison to the standard treatment (dressing alone, hydrocolloid dressing) betulin-based emulsions lead to rapid regeneration of the aesthetic aspects of the skin [30]. For skin regeneration after laser resurfacing, preparations with the *Centella asiatica* extract addition are also used. Their use results in a reduction in skin redness and an improvement in the appearance of the wound. Skin moisture, TEWL, and pH did not differ between the study groups, and their values were, respectively, 34.9 × 0.02 mg/c$m^{2}$, 11.7 g/(h·$m^{2}$) and 5.4 [31]. Another active ingredient that increases the expression of genes key to the healing processes is panthenol. Clinical studies confirm that topical application of panthenol accelerates wound healing, causing rapid re-epithelialization and restoration of skin barrier function after skin injuries [32]. The results of comparative studies of an ointment containing 5% panthenol with petrolatum in wound treatment after laser skin treatment indicate that the ointment with panthenol addition causes faster wound healing and a higher rate of re-epithelialization. Similar results can be achieved with the use of hyaluronic acid [33]. In [34], the effect of a fractional C$O_{2}$ laser in combination with recombinant human epidermal growth factor (rhEGF) on the skin was investigated. The use of rhEGF after C$O_{2}$ laser treatment significantly improves the effectiveness of acne scar treatment, strengthens skin barrier function, and reduces inflammation. An increased water content was observed in the control group from 31 × 0.02 mg/c$m^{2}$ to 34 × 0.02 mg/c$m^{2}$ and in the treatment from 30 × 0.02 mg/c$m^{2}$ to 44 × 0.02 mg/c$m^{2}$ in the stratum corneum, and reduced pH (control group from 5.8 to 5.6, treatment from 5.8 to 5.0) and TEWL (control group from 21 to 19 g/(h·$m^{2}$) treatment from 22 to 16 g/(h·$m^{2}$)) was also observed [34]. Unlike the above ingredients, ectoine has unique properties. As a natural extremolite, ectoine stabilizes biological membranes, protects macromolecules from oxidative stress, and modulates the inflammatory response by reducing the levels of proinflammatory cytokines such as IL-6 and TNF-α. The osmoregulatory and membrane-stabilizing effects of ectoine are particularly beneficial in restoring the integrity of the skin barrier under conditions of extreme stress, such as that induced by ablative fractional laser treatment. While other ingredients mainly focus on hydration or the stimulation of cell proliferation, ectoine offers cytoprotection, preventing structural damage to keratinocytes and maintaining homeostasis in the presence of ROS. All these properties predispose preparations containing ectoine to be used after skin fractionation treatments with a C$O_{2}$ laser. Previous research on ectoine focused mainly on its protective effects in the context of inflammatory skin diseases, atopy, sun damage, and environmental stress. The following paper describes the effect of ectoine on skin parameters, focusing on redness, hydration, and transepidermal water loss (TEWL) after the application of a C$O_{2}$ laser.

## 2. Materials and Methods

### 2.1. Materials

Recipes of three cosmetic emulsions were prepared with identical compositions of base materials with different contents of ectoine as a biologically active raw material. Pharma Cosmetics’ Lekobaza, a traditional product manufactured according to the German monograph DAC (Cremor basalis), was used to make the preparations. Lekobaza is a safe preparation with proven and effective action, containing a minimum amount of ingredients necessary to obtain it. Lekobaza is a preparation with a complex composition, consisting of a mixture of white petroleum jelly with oil-in-water and water-in-oil complex emulsifiers and other ingredients (Table 1). The base is used in the preparation of multiphase ointments, combining the properties of ointments, suspensions, and emulsions in one preparation. It is also used as an additive to creams and liquid emulsions. When applied to the skin, it spreads easily and absorbs quickly. It has a washable base. It moisturizes and nourishes the skin and has a pH value corresponding to the skin’s reaction (pH = 5.5). Due to the water content, it has a cooling effect (water evaporation effect). It is also used as an emulsion base for cosmetic preparations [Fagron materials]. Table 1 below shows the raw materials used in the recipes of the tested emulsions.

### 2.2. Preparation

All the raw materials were weighed on an analytical balance according to recipes A, B, and C in Table 1. The aqueous phase was added to the Lekobaza and mixed with a mechanical stirrer at 2000 rpm for about 20 min. Then, the ready-made preparations were subjected to microbiological and rheological tests. Skin parameters such as pH, redness, hydration, and transepidermal water loss before and after skin fractionation with a C$O_{2}$ laser were also tested. Prepared emulsions A, B, and C were applied to irritated and reddened skin previously fractionated with a C$O_{2}$ laser.

### 2.3. Microbiological Properties

The microbiological purity of the preparations used during the procedure is an important element to prevent postoperative complications. In the test, the active substance ectoine was used in two concentrations, 2% and 5%. Before the use during the treatments, tests were carried out for microbiological purity according to a pharmacopoeial method, in accordance with the requirements of the current Polish Pharmacopoeia (FP XI) and the European Pharmacopoeia. The study was guided by the acceptance criteria for this group of preparations, which consist of the assessment of the TAMC (Total Aerobic Microbial Count) and the TYMC (Total Yeast/Mold Count). The standards for non-sterile preparations applied to the skin in the TAMC test allow 10^2^ CFU/g and 10^1^ CFU/g in the TYMC test. Examinations were carried out for the following:

1. The total number of microorganisms—direct culture.
2. The presence of *Staphylococcus aureus*, *Escherichia coli*, and *Pseudomonas aeruginosa* bacteria—at 0.1 g/mL.
3. Total amount of fungi (yeasts, molds)—direct inoculation.
4. The number of *Candida albicans* fungal cells—in 0.1 g/mL.

This study used Columbia Agar +5% sheep blood, Mueller Hinton E, Sabouraud Gentamicin Chloramphenicol 2 Agar, and Trypcase–soy broth liquid medium to dilute the test samples, from bioMerieux Polska Sp. z o.o. The test samples in the amount of 0.1 g and the serial dilutions prepared from them (1:10, 1:100, and 1:1000), in the amount of 0.1 mL, were applied to 2 plates of each type of medium (direct inoculation method—surface inoculation). Incubation was carried out for 5 days at 35 °C (TAMC) and for 7 days at 25 °C (TYMC). The results are the arithmetic mean of the number of colonies for plates of different dilutions.

### 2.4. Application of C$O_{2}$ Fractional Laser and Measurement of Skin Parameters

Lasers used in cosmetology aim to eliminate a cosmetic defect, such as scars, which are removed by fractionation of the skin, causing long-term skin discomfort by weakening its external barrier, and thus exposing the skin to the negative influence of the external environment. The return of the skin to the state it was before the treatment is a long process [35]. The use of ectoine shall allow us to achieve the same therapeutic effect with much less discomfort and to maintain better skin parameters, which allow for a more effective course of its repair processes. The light of the fractional C$O_{2}$ laser penetrates deep into the skin and is absorbed by the water contained in the cells. As a result of the photothermal reaction, the water in the tissue is rapidly heated, then evaporated, and heat is released, which leads to micro-damage to collagen and fractional disruption of skin continuity. At the same time, repair processes are initiated, which initiate the renewal of damaged structures. The aim is to damage the skin and then regenerate it, which leads to smoothing after the healing process is completed. The fractional laser works based on the phenomenon of fractional photothermolysis. This method consists of creating microscopic, non-adherent columns of micro-damage in the skin, surrounded by an area of living, undamaged tissue. Therefore, the device does not damage the skin in its entirety, but only “punctures” it. Thanks to this, the skin regenerates faster after the procedure, significantly shortening the healing time and reducing the risk of complications. A Yasumi C$O_{2}$ fractional laser was used during the treatment. Classic laser operating parameters were used, for a person with white skin on the Fitzpatrick II scale. The applied initial operating parameters are safe for all subjects with this type of skin. During the procedure, the operating parameters of the device presented in Table 2 were applied.

During the process of qualifying persons admitted to the research, the following exclusion criteria were used: skin diseases, general poor health, pregnancy and breastfeeding, skin cancer, and hypertrophic scars. The test fields were determined as follows:

1. Marking of the collarbone from the side of the breastbone.
2. Connection of the middle point of the collarbone with a horizontal line.
3. Marking a vertical line 120 mm long from the center of the line joining the collarbones.
4. Dividing the vertical line every 40 mm.
5. Obtaining test fields with dimensions of 40 × 40 mm.

The test fields are marked as P1, P2, P3, P4, P5, and P6, respectively, and are shown in the Table 3.

In accordance with the marking of the test areas after the C$O_{2}$ laser treatment, preparations A, B, and C were applied in individual areas in accordance with the scheme described above. Thirty people aged eighteen to sixty-five, who had no skin diseases and who had already undergone laser treatments in the past, were qualified for this study. People participating in the research were informed about the treatment restrictions, the procedure, and post-treatment recommendations as part of the research. All subjects were subjected to the same skin parameter measurement procedure:

1. Before starting laser therapy.
2. 48 h after laser therapy.
3. 7 days from the first application of the tested preparations.
4. 14 days from the first application of the tested preparations.
5. 21 days from the first application of the tested preparations.
6. 28 days from the first application of the tested preparations.

The following skin parameters were tested: hydration, redness, pH, and transepidermal water loss. During the tests, the Courage & Khazaka (Cologne, Germany) Multi Probe Adapter MPA System was used, with the following probes: the Corneometer^®^ CM 825 Probe, Skin-pH- Meter^®^ PH 905 Probe, Mexameter^®^ MX 18 Probe, and Tewameter^®^ TM 300 Probe. For each subject, the test was performed three times, applying the probe to non-overlapping areas of the skin every 20 s, and the result was recorded as the arithmetic mean of the obtained measurements. The ambient conditions during the measurements were as follows: temperature 20–22 °C, humidity 40–60%, which were within the ranges specified by the manufacturer of the device. Skin hydration was measured with a Corneometer^®^ CM 825 Courage & Khazaka (Cologne, Germany). One-second measurements were made by applying a probe to the selected area of the body, which measures 10–20 μm into the stratum corneum. The measurement is based on the assessment of the skin’s electrical capacity, the values of which are directly related to the state of skin hydration. The results are given in conventional units of the corneometer, specified by the equipment manufacturer, in which 1 unit corresponds to 0.02 mg of water per c$m^{2}$ of the stratum corneum. The lower the result, the more the skin’s outermost layer (20 μm) is dehydrated. Usually, a normal skin hydration level is considered to be >40 units, 30–40 units is dry skin, and <30 units is very dry, dehydrated skin [27]. Skin pH measurements were made using the Skin-pH-Meter^®^ PH 905 probe, which measures the acid–base pH of the skin. The probe was calibrated before the measurement. Melanin levels and skin redness were measured with the Mexameter^®^ MX 18 Probe, the Courage & Khazaka (Cologne, Germany). These two parameters are the factors with the highest influence on skin color. Measurement is based on the absorption/reflection principle. The probe emits light at three specific wavelengths (the manufacturer does not specify the range in nanometers). The receiver measures the light reflected by the skin. Tewameter^®^ TM 300, the Courage & Khazaka (Cologne, Germany) measures the level of transepidermal water loss through the skin, which allows us to determine the degree of skin hydration or the state of skin barrier function. Each subject was tested in triplicate, applying the probe to non-overlapping skin areas every 20 s, and measurements were made in triplicate in each of the designated test areas. The test environment was a room with constant ambient conditions: temperature 20 °C to 22 °C, humidity at the level of 40–60%, and sunlight limited by a low-transmittance roller shutter. Measurements were taken at 15:00 in the same lighting conditions.

### 2.5. Rheological Measurements

Testing the rheological properties of cosmetic preparations, including emulsions, is an important determinant of the quality of cosmetic preparations. Often, rheological studies also replace sensory evaluation, texture and chemical analysis of some cosmetics. The rheological properties of the emulsion were tested at 4 °C, 20 °C, 32 °C (human skin temperature when applying the cosmetic preparation), and 40 °C. A Haake RS-6000, (ThermoFisher, Karlsruhe, Germany) rotational rheometer was used for the tests, using a cone–plate system with a diameter of 35 mm, angle 2° and a gap of 0.2 mm. Rheological tests of the obtained cosmetic preparations were carried out immediately after their preparation and after a 2-month storage period. The tests were carried out with an increasing shear rate in the value range from 1 $s^{-1}$ to 1000 $s^{-1}$. The Cross model, expressed by Equation (1), was used to describe the rheological properties.

(1) $$\eta =\eta_{\infty }+\frac{\eta_{0}-\eta_{\infty }}{1+K{\left(\dot{\gamma }\right)}^{n}}$$

where $\eta_{0}$—the apparent viscosity at zero shear rate, Pa·s; $\eta_{\infty }$—apparent viscosity for infinite shear rate, Pa·s; $\dot{\gamma }$—shear rate, $s^{-1}$; *n*—flow behavior index; and *K*—relaxation time, s. The estimation of the parameters of rheological models containing time constants allows for determining the most probable characteristic time of the tested material. Determining the changes in the values of the constants in the Cross equations as a function of concentration allows for the formation of a view of the structure of rheological phenomena occurring during shearing. The basic criterion selection of the vibration amplitude value involves carrying out the measurement in the range of linear viscoelasticity (LVE), then measurements are performed at a constant frequency value, determining the relationship between the storage modulus $G^{\prime }$ (Pa) and the loss modulus $G^{\prime \prime }$ (Pa) as a function of the deformation amplitude. The range of linear viscoelasticity is defined in the region of the experimental curves independent of the applied strain amplitude. Determining the appropriate value most often involves determining the maximum amplitude in the LVE range, which translates into the greatest possible deformation and thus increases the accuracy of the measurements. The range of linear viscoelasticity was determined by determining the dependence of the storage modulus $G^{\prime }$ and the loss modulus $G^{\prime \prime }$ at a constant frequency of 1 Hz. The frequency sweep test was performed in the range of linear viscoelasticity at a constant strain in the frequency range of 0.1 Hz to 100 Hz. The method of determining the plasticity limit consisted of subjecting the test material to stresses that increased linearly over time and observing the deformation. The value of the plasticity limit ($\tau_{0}$) is defined as a point where two defined lines intersect in the $log(\tau_{0})$-$log(\gamma_{0})$ coordinate system. The point is characterized by a curvature of Relation [36]. For all cases, the range of stress applied was determined, and the entire analysis was carried out within 300 s.

### 2.6. Statistical Analysis

In the statistical analysis, Student’s *t*-test (dependent *t*-test) was used. Statistical analysis was performed using Statistica 13.

## 3. Results and Discussion

### 3.1. Microbiological Analysis

The preparations used in this research are based on the commercial raw medical materials and preparations approved and certified for use by humans and were subjected to microbiological tests. In the conducted microbiological tests, after incubation, no growth of mesophilic microorganisms was obtained in any of the dilutions; therefore, the tested ectoine samples meet the requirements for the preparations and cosmetics applied to the skin in accordance with the Polish Pharmacopoeia XI edition, Supplement 2019 to the Polish Pharmacopoeia XI edition (Supplement 2019 FP XI) and European Pharmacopoeia (Ph. Eur.) 10th Edition.

### 3.2. Application of C$O_{2}$ Fractional Laser and Measurement of Skin Parameters

The important properties of cosmetic emulsions are their protection against stress factors that may lead to dehydration. Dry air, especially in periods of frost or heat and air conditioning, tends to dry out the skin significantly. In order to demonstrate the protective effect of ectoine on skin hydration, cosmetic preparations were applied with various contents of ectoine and without it. The preparations were applied topically as shown in Table 3 for 1 month. Skin moisture was determined by the corneometric method, and the results are presented in Table 2. Corneometer measurements in the case of preparation C showed no significant differences (NS) between the tests before and one month after the procedure. Statistically significant differences between the studies were found in the cases of preparation B (*p* < 0.001) and preparation A (*p* < 0.001) applied once a day and preparation A applied twice a day. There were no statistically significant differences between the studies in both control groups, the first after the laser (CwL) and the second without the laser (CnL). The analysis of changes between the tests before and after one month (CBT) from the procedure observed with the corneometer showed a statistically significant (*p* < 0.001) increase in the value after the application of preparation B. The differences in the observed changes between the use of preparation B and preparation A increased from 7.7 to 14.4 and were significant at *p* < 0.001. Administration of formulation A twice a day resulted in a significant increase in the observed changes between studies (*p* < 0.001) from 14.4 for formulation A applied once a day to 18.1 for formulation A applied twice a day. The changes observed between the tests before and after the month (a1m) were significantly (*p* = 0.037) higher for the control sample without any preparation (CnA). Comparison of the results of the study one month after corneometer treatment with the control without any interference (pa1m/CnL) indicates the presence of significantly higher values for preparation B (*p* < 0.001), preparation A (*p* < 0.001), and preparation A applied twice a day (*p* < 0.001). The tests performed one month after the procedure did not differ significantly from the control without any interference for preparation C and for the control sample without preparations. The results show that the ectoine contained in the cosmetic oil-in-water emulsion protects the skin against dehydration, providing a higher moisture content than the basic formulation (placebo). Ectoine protects the skin against dehydration that occurs after laser treatment. Ectoine, thanks to its ability to strongly bind water molecules, leads to the formation of a water layer not only around itself but also around neighboring molecules. In the presence of ectoine, water molecules take on a more compact structure. The skin’s moisture can be maintained for a long time by applying ectoine topically. Merck presents the results of similar studies at different concentrations of ectoine addition to the cream on skin hydration levels. The presented results indicate a significantly higher moisture content of the skin treated with the cream with ectoine addition compared to the control group. The test results are summarized in Table 4.

### 3.3. pH Measurement

Measurements with a pH meter showed the existence of statistically significant differences between the studies in the case of preparation C (*p* = 0.009), preparation B (*p* < 0.001), preparation A (*p* < 0.001), and twice-daily preparation A. No statistically significant differences between the tests occurred in the control group without any interference. The analysis of pH changes between the tests before and one month after the treatment showed a significant increase in pH between preparation C and preparation B. Increasing the dose of preparation A did not increase the pH. The observed pH change was the same as for formulation B. Twice-daily administration of preparation A resulted in a significant (*p* < 0.001) increase in pH from 0.2 to 0.5. The changes in pH observed between the tests before and after one month differed significantly (*p* = 0.002) between preparation C and the control group without any interference (CnA). Comparison of the results of the pH test one month after the procedure with the control without any interference indicates the presence of a significantly lower pH for preparation B (*p* = 0.001), preparation A (*p* < 0.001), and preparation A applied twice a day (*p* < 0.001), and a higher pH for the control group without application (*p* < 0.001). The pH tests performed one month after the treatment did not differ significantly from the control without any interference for preparation C. The test results are summarized in Table 5.

### 3.4. TEWL Measurement

TEWL measurements showed statistically significant differences between the tests for preparation C (*p* = 0.005), preparation A (*p* < 0.001), preparation A twice daily (*p* < 0.001), and the laser control (*p* < 0.001). There were no statistically significant differences between the tests for preparation B and for the control group without a laser. Changes between the tests before and one month after the procedure, measured with the tevameter, show a continuous significant decrease in these changes starting with the administration of preparation B (*p* = 0.006), preparation A applied once a day, and preparation A applied twice a day (*p* < 0.001). The observed changes between the tests are significantly (*p* < 0.001) higher for the control group without a laser than for preparation C. Comparison of the results of the tevameter test one month after the procedure with the control group without any interference indicates the presence of significantly lower values for preparation B (*p* = 0.016), once-a-day preparation A (*p* < 0.001), and twice-a-day preparation A (*p* < 0.001), and higher for the no-application control (*p* < 0.001). Tevameter tests performed one month after the procedure did not differ significantly between the control group without any application and preparation C. The lowest parameter values for the skin after laser treatment and after the application of preparation A, and the highest for the skin after C$O_{2}$ laser treatment and without application of any preparations, were obtained. The results are summarized in Table 6. The use of an emulsion containing various amounts of ectoine leads to a reduction in TEWL; the best results were obtained using the preparation with 5% ectoine content, twice a day. A similar reduction in TEWL was demonstrated in [17], where an oil-in-water emulsion containing 0%, 2%, and 5% of ectoine was applied. Previously, the skin was damaged with a sodium dodecyl sulfate (SDS) solution to increase TEWL. The change in TEWL values before and after the application of the emulsion containing ectoine and after damage to the skin barrier with SDS was investigated.

### 3.5. Mexametry

Measurements with the mexameter with erythema showed a statistically significant increase in the measured values between the tests for preparation C (*p* < 0.001), preparation B (*p* < 0.001), preparation A applied once a day (*p* < 0.001), preparation A applied twice a day, and for the control group with a laser (*p* < 0.001). There were no statistically significant differences between the tests in the control group without the laser. Changes between the tests before and one month after the procedure, measured with a mexameter, indicate a continuous significant decrease in these changes, starting with the administration of preparation B (*p* < 0.001), preparation A applied once a day (*p* < 0.001), and preparation A twice a day (*p* < 0.001). The observed changes between the tests are significantly (*p* < 0.001) higher for the control group without a laser than for preparation C. Comparison of the results of the mexameter test one month after the procedure with the control test without any interference indicates the presence of significantly lower values for preparation C (*p* < 0.001), preparation B (*p* < 0.001), preparation A applied once a day (*p* < 0.001), and preparation A applied twice a day (*p* < 0.001), and higher for the control without any application (*p* < 0.001). The lowest parameter values for the skin after laser treatment and after application of preparation A, and the highest for the skin after C$O_{2}$ laser treatment and without application of any preparations, were obtained. The results are summarized in Table 7.

The use of a cosmetic emulsion containing various amounts of ectoin addition leads to a significant reduction of TEWL to 15% (Table 6), a reduction of redness by 30% (Table 7), and an increase in the degree of hydration by 30% (Table 4). Correct skin parameters such as pH, moisture levels, or correct TEWL levels are conditions for good and effective skin regeneration without disturbing its healing process. An increase in the TEWL level, and thus a decrease in hydration, weakens the epidermal barrier and disrupts the extracellular matrix (ECM). Additionally, a change in pH—an increase—would have a direct effect on the development of bacteria living on the skin. In summary, thanks to the use of ectoine, correct skin parameters were maintained, which contributed directly to effective and proper skin regeneration without side effects.

### 3.6. Rheological Studies

**Figure 2:**
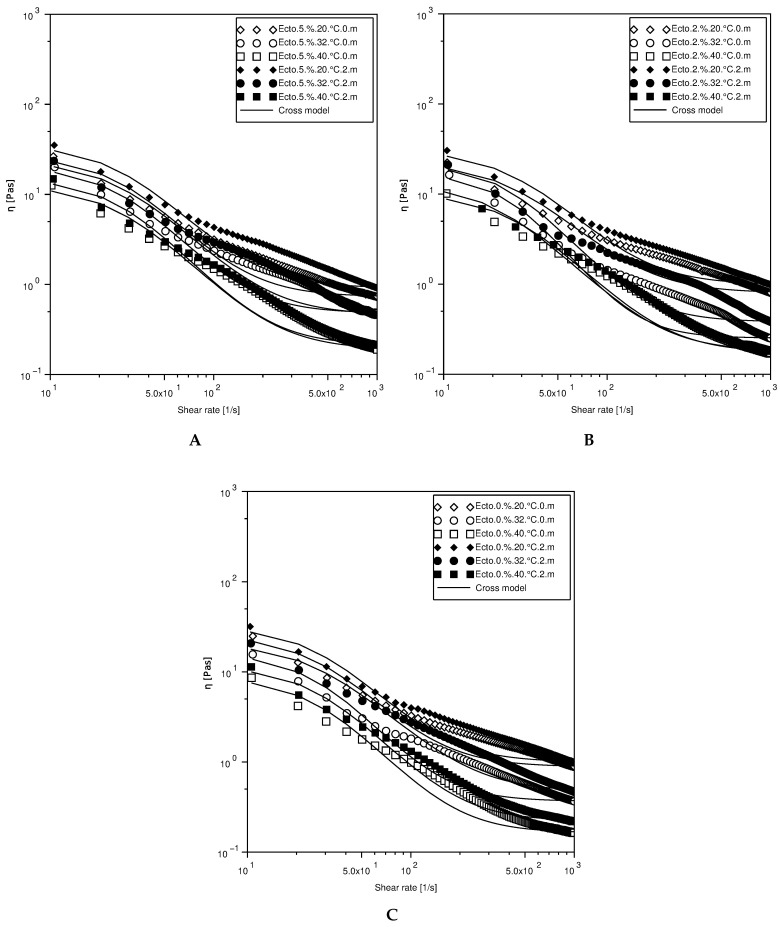
Viscosity flow curves for emulsions A, B, and C at 20, 32, and 40 °C, immediately after receiving the emulsion and after a two-month storage period.

**Figure 3:**
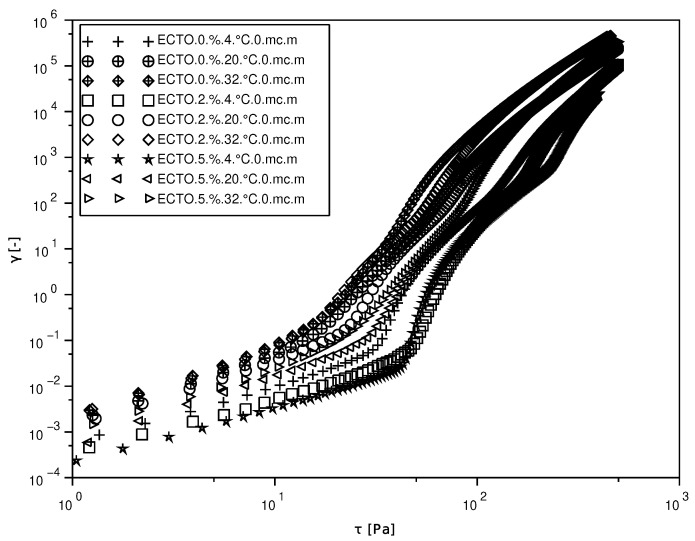
Yield stress for emulsions A, B, and C at 4, 20, and 32 °C.

**Figure 4:**
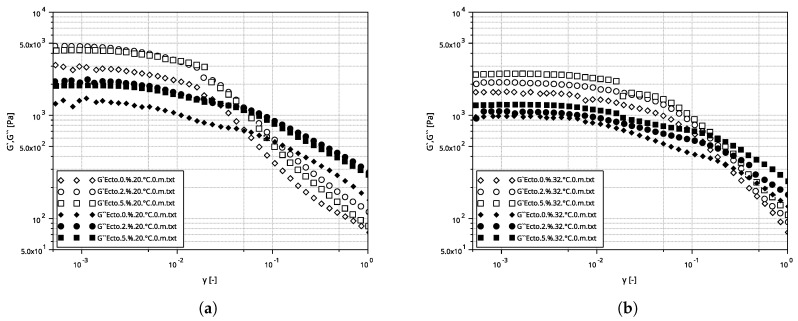
Relationship between the $G^{\prime }$ and $G^{\prime \prime }$ modulus values as a function of strain amplitude for emulsions A, B, and C at temperatures of (**a**) 20 °C, and (**b**) 32 °C.

**Figure 5:**
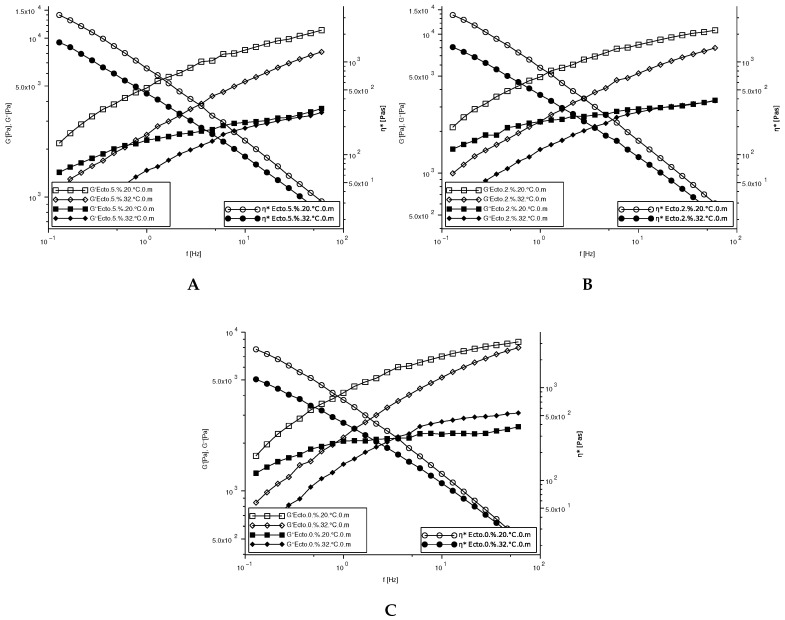
$G^{\prime }$ and $G^{\prime \prime }$ as a function of frequency for emulsions A, B, and C at 20 °C and 32 °C.

The results of rheological tests of the obtained preparations are presented graphically in Figure 2, Figure 3, Figure 4 and Figure 5. The analysis of the experimental data is presented in Figure 2 concerning the viscosity flow curves of cosmetic emulsions. The experimental data were fitted to the Cross model, and the variability of the rheological parameters K and n was calculated and is presented in Table 8.

Figure 2 shows the viscosity flow curves for emulsions A, B, and C at 20 °C, 32 °C and 40 °C. The analysis of the experimental data presented in Figure 2 concerning the viscosity flow curves of cosmetic emulsions allowed us to conclude that the preparations in the analyzed range of shear rate at all tested temperatures are non-Newtonian shear-diluted fluids with a flow limit (the shear-thinning phenomenon). The apparent viscosity of tested emulsions decreases in the range of 35–0.2 Pa·s. With an increase in the temperature, the apparent viscosity decreases for all tested systems. In the case of preparation A, the apparent viscosity was the highest, and the lowest was for preparation C. With an increase in the ectoine content in the preparation, an increase in viscosity was observed. After a two-month storage time, the viscosity flow curves for temperatures of 20 and 32 °C show that viscosity values and the shear rate are similar for preparations B and C. The viscosity is almost identical for the preparations at 40 °C.

The flow limit is a parameter tested in the case of ointments and creams for topical use, important when applying the preparation to the skin [37]. The acceptance of pharmaceutical and cosmetic products by consumers depends on their sensory experience from the moment they are taken out of the container to when they are applied to the skin. The storage stability and sensory properties of these products are determined by their rheological properties, especially the size of the yield point. A high value of the yield point may make it difficult to distribute the product on the skin. And skin that is red or irritated due to fractionating with a C$O_{2}$ laser may feel discomfort or even pain when applying and spreading the preparation. Figure 3 illustrates the effect of temperature and ectoine concentration on the yield stress values of the tested preparations. The yield stress values for preparations A, B, and C at temperatures of 4, 20, and 32 °C are shown. The highest value of $\tau_{0}$ at a temperature of 4 °C was observed for preparation B. Preparation A shows the highest value of $\tau_{0}$ at temperatures of 20 °C and 32 °C.

An increase in deformation is visible after exceeding the stress level for emulsions A, B, and C, with values of 50 Pa, 45 Pa and 31 Pa, respectively, at a temperature of 4 °C. The addition of ectoine caused a slight increase in the yield stress, while the increase in temperature caused a decrease. For example, for emulsion A, the increase in temperature caused the yield stress to decrease from 45 Pa to 20 Pa.

In Figure 4, the storage modulus $G^{\prime }$ and loss modulus $G^{\prime \prime }$ as a function of deformation for the tested emulsions A, B, and C at temperatures of (a) 20 °C and (b) 32 °C are shown. At low strain amplitudes, $G^{\prime }$ is almost constant, suggesting a linear viscoelastic range where no permanent damage to the sample structure occurs. Then, both moduli become strain-dependent, with both $G^{\prime }$ and $G^{\prime \prime }$ decreasing and passing through an intersection point, the strain of which is defined as the failure strain [38].

The viscoelastic properties of emulsions A, B, and C at 20 °C and 32 °C were determined using dynamic properties, and the storage modulus $G^{\prime }$, loss modulus $G^{\prime \prime }$, and complex viscosity $\eta^{*}$ are given in Figure 5. The $G^{\prime }$ values are a measure of the strain energy stored in the sample of the preparation during the shearing process, representing the elastic behavior of the preparation sample. The $G^{\prime \prime }$ value is a measure of the strain energy used in the sample during shearing and lost by the sample afterwards [39]. If $G^{\prime }$ is much larger than $G^{\prime \prime }$, the material will behave more like a solid; that is, the deformations will be essentially elastic or recoverable. However, if $G^{\prime \prime }$ is much larger than $G^{\prime }$, the energy used to deform the material is dissipated viscously and the material behaves similarly to a liquid [38]. The behavior of the tested emulsions is clearly more elastic than viscous, since in all cases, $G^{\prime }$ dominates over $G^{\prime \prime }$ over the full frequency range. $G^{\prime }$ and $G^{\prime \prime }$ increase with increasing frequency without any crossing point; this indicates that the elastic character of the emulsion dominates over the viscous character in the tested conditions, and similar behavior of the curves is observed in the work [40]. This elastic behavior is a consequence of the repulsive forces between the droplets, which are magnified at such a high droplet concentration. This type of mechanical spectrum is characteristic of weak gels [41]. Moreover, an increase in the difference between $G^{\prime }$ and $G^{\prime \prime }$ is observed with increasing frequency, which indicates a decrease in the tan(δ) value and therefore an increase in the elastic values of the tested emulsions. The addition of ectoine slightly increases the values of $G^{\prime }$ and $G^{\prime \prime }$.

The $G^{\prime }$ and $G^{\prime \prime }$ values obtained for 20 °C and 32 °C are similar; they increase as a function of frequency. The increase in temperature leads to a marked decrease in the value of the modules. The tests of viscoelastic properties in the linear range are carried out under low-deformation conditions, which allows the observation of the structural features of the emulsion.

## 4. Conclusions

In this work, the influence of ectoine addition to cosmetic preparations on skin parameters after fractionation treatment with a C$O_{2}$ laser was examined. A unique role of ectoine in the prevention of transepidermal water loss caused by skin damage by standard C$O_{2}$ laser treatment has been shown. Ectoine acts as a stronger moisturizing agent and provides long-term skin moisturizing effectiveness. With an increase in the percentage of ectoine and the frequency of application, the occurrence of redness after C$O_{2}$ therapy decreased, and the best results were obtained with preparation A applied twice a day. TEWL decreased between the tests before and after one month after the treatment; the best result was obtained for preparation A applied twice a day from 6.2 to 5.3 g/($m^{2}$·h). pH values also decreased, with the greatest decrease from 4.8 to 4.3. The highest increase in skin hydration level values was obtained for preparation A and ranged from 54.4 × 0.02 mg/c$m^{2}$ to 72.5 × 0.02 mg/c$m^{2}$. The results of rheological tests of the tested preparations allowed us to conclude that the preparations in the analyzed shear rate range at all tested temperatures are non-Newtonian fluids that are shear-thinning and have a flow limit. The highest yield stress values at 20 °C for formulations A at 32 Pa and B at 25 Pa were obtained. The strong water-binding action of the ectoine may be particularly useful in preventing water loss from dry atopic skin and can assist in treatment of the damaged skin after laser procedures. Ectoine addition to cosmetic preparations ensures better wound healing results and reduces undesirable complications, among other benefits. 

## Figures and Tables

**Table 1:** Raw materials included in the recipes of tested A, B, and C emulsions.

No.	INCI Ingredients	Producer	A [g]	B [g]	C [g]
	Components of Lekobaza [Fagron materials]:				
	Glyceryl Stearate (4.0 g)				
	PEG-20 Glyceryl Stearate (7.0 g)				
1	Caprylic/Capric Triglyceride (7.5 g)	Fagron sp. z o.o	85	88	90
	Cetyl Alkohol (6.0 g)	(Kraków,			
	Petrolatum (25.5 g)	Poland)			
	Propylene Glycol (10.0 g)				
	Aqua, Water (40.0 g)				
2	Ectoin-RonaCare^®^Ectoine	Merck	5	2	-
		(Darmstadt,			
		Germany)			
3	Aqua, Water	Fagron	to 100	to 100	Do 100

**Table 2:** The applied parameters of the C$O_{2}$ laser.

No.	Name	Value
1	Point Energy [mJ]	25.0 with Coverage Rate 50%
2	Power [W]	25
3	Duration [ms]	1.0
4	Interval [ms]	1.0
5	Density [mm]	0.5
6	Overlap [th]	1

**Table 3:** Location of the test areas on the test person.

Test Area	Control Areas			Description
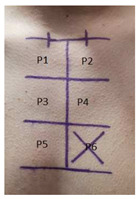	P1, P2, P3, P4, P5			Deliberate damage to the epidermis with a C$O_{2}$ laser.
	P6			Control area.
	P1			Preparation C applied once a day.
	P2			Preparation B applied once a day.
	P3			Preparation A applied once a day.
	P4			No application of the preparation.
	P5			Preparation A applied twice a day.
	P6			Control area.
Marked test areas				

**Table 4:** Results of the corneometry measurements.

	K [0.02 mg/c$m^{2}$]						
Variable	Before	After Month	$p_{M}$	CBT	$p_{\mathrm{CBT}}$		$p_{a1m/\mathrm{CnL}}$
Preparation A twice a day	54.4	72.5	<0.001	18.1	<0.001		<0.001
Preparation A once a day	53.9	68.3	<0.001	14.4		<0.001	<0.001
Preparation B once a day	54.2	61.9	<0.001	7.7	<0.001		<0.001
Preparation C once a day	53.9	54.6	NS	0.7		=0.037	NS
CnA	54.4	53.6	NS	−0.8			NS
CnL	54.6	54.7	NS	-	-	-	-
CwL	54.4	53.6	NS	-	-	-	-

$p_{M}$—significance level before–after one month; NS—no significant differences; CBT—changes between the tests before and after one month; $p_{CBT}$—significance level for changes between the tests before and after one month; CnA—control without application; CnL—control without laser; CwL—control with laser.

**Table 5:** Results of the pH-metry.

	pH						
Variable	Before	After 1 Month	$p_{M}$	CBT	$p_{\mathrm{CBT}}$		$p_{a1m/\mathrm{CnL}}$
Preparation A twice a day	4.8	4.3	<0.001	0.5	<0.001		<0.001
Preparation A once a day	4.7	4.5	<0.001	0.2		NS	<0.001
Preparation B once a day	4.8	4.6	<0.001	0.2	<0.003		=0.001
Preparation C once a day	4.8	4.7	<0.009	0.1		<0.002	NS
CnA	4.8	4.9	<0.001	−0.1			<0.001
CnL	4.7	4.7	NS	-	-	-	-
CwL	4.8	4.9	<0.001	-	-	-	-

$p_{M}$—significance level before–after one month; NS—no significant differences; CBT—changes between the tests before and after one month; $p_{CBT}$—significance level for changes between the tests before and after one month; CnA—control without application; CnL—control without laser; CwL—control with laser.

**Table 6:** Results of the TEWL measurements.

	T [g/($m^{2}$·h)]						
Variable	Before	After Month	$p_{M}$	CBT	$p_{\mathrm{CBT}}$		$p_{a1m/\mathrm{CnL}}$
Preparation A twice a day	6.2	5.3	<0.001	−0.9	<0.001		<0.001
Preparation A once a day	6.1	5.7	<0.001	−0.4		<0.001	<0.001
Preparation B once a day	6.0	6.0	NS	0	=0.006		=0.016
Preparation C once a day	5.9	6.2	=0.005	0.3		<0.001	NS
CnA	6.1	7.7	<0.001	1.6			<0.001
CnL	6.3	6.3	NS	-	-	-	-
CwL	6.1	7.7	<0.001	-	-	-	-

$p_{M}$—significance level before–after one month; NS—no significant differences; CBT—changes between the tests before and after one month; $p_{CBT}$—significance level for changes between the tests before and after one month; CnA—control without application; CnL—control without laser; CwL—control with laser.

**Table 7:** Results of the mexametry.

	M [-]						
Variable	Before	After Month	$p_{M}$	CBT	$p_{\mathrm{CBT}}$		$p_{a1m/\mathrm{CnL}}$
Preparation A twice a day	347	373	<0.001	26	<0.001		<0.001
Preparation A once a day	344	425	<0.001	81		<0.001	<0.001
Preparation B once a day	347	466	<0.001	119	<0.001		<0.001
Preparation C once a day	346	489	<0.001	143		<0.001	<0.001
CnA	345	523	<0.001	178			<0.001
CnL	347	347	NS	-	-	-	-
CwL	345	523	<0.001	-	-	-	-

$p_{M}$—significance level before–after one month; NS—no significant differences; CBT—changes between the tests before and after one month; $p_{CBT}$—significance level for changes between the tests before and after one month; CnA—control without application; CnL—control without laser; CwL—control with laser.

**Table 8:** Cross viscosity rheological model parameters.

Preparation	T [°C]	$\eta_{0}$ [Pa·s]	$\eta_{\infty }$ [Pa·s]	K [s]	n	$R^{2}$
A 5% 0.m	20	26.37	0.73	0.0012	2.047	0.980
	32	20.10	0.48	0.0011	2.080	0.980
	40	12.63	0.19	0.0016	1.961	0.980
B 2% 0.m	20	22.41	0.80	0.0020	1.888	0.980
	32	16.38	0.25	0.0004	2.438	0.978
	40	10.16	0.17	0.0020	1.965	0.979
C 0% 0.m	20	24.97	0.89	0.0014	2.006	0.973
	32	15.64	0.37	0.0010	2.173	0.975
	40	8.59	0.16	0.0011	2.082	0.976
A 5% 2.m	20	35.16	0.91	0.0014	1.990	0.991
	32	23.50	0.46	0.0017	1.940	0.992
	40	14.88	0.21	0.0011	2.074	0.992
B 2% 2.m	20	30.48	0.99	0.0017	1.934	0.985
	32	21.18	0.38	0.0008	2.199	0.985
	40	15.62	0.18	0.0023	1.989	0.983
C 0% 2.m	20	31.85	0.99	0.0014	1.993	0.984
	32	20.77	0.47	0.0021	1.848	0.984
	40	11.39	0.22	0.0011	2.057	0.984

## Data Availability

Data are contained within the article.
